# Patterns and characteristics of polypharmacy among elderly residents in Danish nursing homes

**DOI:** 10.2144/fsoa-2020-0039

**Published:** 2020-05-29

**Authors:** Jacob Astorp, Mimoza Gjela, Pernille Jensen, Rasmus D Bak, Parisa Gazerani

**Affiliations:** 1 Department of Health Science & Technology, Faculty of Medicine, Aalborg University, 9220 Aalborg E, Denmark

**Keywords:** drug, drug–drug interaction, elderly, multimorbidity, nursing homes, polypharmacy

## Abstract

**Aim:**

To identify patterns and characteristics of polypharmacy among elderly residents in Danish nursing homes in the Northern region of Denmark.

**Materials & methods:**

Twenty-five nursing homes were contacted, where each supplied 20 randomly selected anonymized residents’ information. Residents were 65 years or older, concurrently taking five or more medications. Drug–drug interactions and potential adverse effects were investigated.

**Results:**

One hundred residents (68% females; 32% males) were included. The most prevalent co-morbid condition was cardiovascular disease, and the most prevalent medications were for gastrointestinal- and metabolism-related conditions. Age influenced the number of drugs (p = 0.013) and drug–drug interactions per resident (p = 0.039), with a positive correlation.

**Conclusion:**

Elderly residents of the studied nursing homes were potentially affected by an inappropriate polypharmacy.

## Lay abstract

Multimorbidity is common among elderly and a leading factor for polypharmacy. We conducted this study on 100 Danish elderly residents (>65 years, on ≥5 medications) in 25 nursing homes, anonymously and randomly chosen in Northern region of Denmark, to find characteristics of potential inappropriate polypharmacy. We found that age influenced the number of drugs (p = 0.013) and number of drug–drug interactions per resident (p = 0.039), but no sex-related difference was evident. Positive correlations were present between the number of drugs and drug–drug interactions. Elderly residents of the studied nursing homes were potentially affected by an inappropriate polypharmacy.

The percentage of the population above the age of 60 years old has increased drastically throughout the last few decades. It is estimated that a twofold increase of the world’s elderly population will occur by 2050, reaching nearly 2.1 billion people aged 60 or above [1]. Age has been demonstrated to have an evident association with multimorbidity [2], which is the coexistence of multiple long-term health conditions in a person [3]. It is estimated that up to 95% of the population aged 65 years or older are affected by multimorbidity [3]. In a study performed in a Swedish nursing home, residents exhibited on average 16.8 ± 5.3 separate chronic health problems [4]. The top ten multimorbidities have been reported as hypertension, Type 2 diabetes, depression, lipid disorders, chronic obstructive pulmonary disease, ischemic heart disease, atrial fibrillation, obesity, asthma and dementia [5]. The most common affected organ systems have been identified as cardiovascular system followed by the central nervous system (CNS). Accordingly, the three most commonly prescribed drugs have been recorded as psycholeptics, cardiac medications and diuretics [6].

Multimorbidity results in an increased risk of hospitalization, loss of physical functioning, depression, premature death and polypharmacy [3,7,8]. No consensus currently exists for definition of polypharmacy. A systematic review of 110 studies on polypharmacy has stated that the most commonly used definition is based on concomitant consumption of five or more (>5) drugs [8], which has been reported in 44% of individuals aged 65 years or above [9]. Polypharmacy, is associated with higher risk of adverse outcomes such as frailty, disability, mortality and falls [10]. In one hand, polypharmacy seems necessary to manage multimorbidity in the elderly to target pathogeneses of a diverse range of diseases [11]. On the other hand, risks may arise when inappropriate polypharmacy exists, which is defined as an irrational prescription of too many drugs [12]. Appropriate polypharmacy occurs when the decisions concerning the prescription of multiple drugs to one patient rely on evidence-based practices, achieving the desired clinical effect without compromising the patient’s quality of life by acquiring a reduction in drug toxicity [13]. However, inappropriate polypharmacy occurs when the prescription of multiple drugs to one patient fails to achieve the desired therapeutic effect, only resulting in increased risks and harmful health outcomes [13].

Inappropriate polypharmacy is a worldwide problem [12,14], including Scandinavian countries. For instance, Nyborg *et al.* [15] investigated use of potentially inappropriate medications (PIMs) among elderly living in nursing homes in Norway and demonstrated that 43.8% of the residents were prescribed at least one PIM, while 9.9% were treated with at least three PIMs. The prevalence of PIMs was also higher among females [15]. Inappropriate polypharmacy results in an increased risk of avoidable negative health outcomes [13] and has been associated with higher risks of adverse drug events (ADEs) [16], drug interactions, for example, drug–drug interactions (DDIs) [17], medication nonadherence and functional decline [18]. A subcategory of ADEs are adverse drug reactions (ADRs), defined as harmful or unpleasant reactions related to use of drugs [19]. Hanlon *et al.* [20] determined that polypharmacy consistently increases the risk of ADRs in elderly patients. In particular, it has been reported that this issue is higher in nursing homes [13], and patients on nine or more drugs are more than twice as likely to experience an ADR than those receiving less than nine [21]. This poses a serious problem because ADRs are difficult to distinguish from symptoms that are already present in the elderly, such as delirium and falls [22], making it difficult to choose the correct treatment, which can possibly lead to further inappropriate prescriptions [14]. Furthermore, the risk of hospitalization in the elderly with polypharmacy rises through an increase in ADEs [23]. Managing polypharmacy in the elderly, therefore, can be very challenging [22] and time consuming [24]. It is because of age-related changes in the physiology of the elderly population that cause alterations in drug disposition and metabolism [25–27], one example is the decline in the capacity of Phase I reaction enzymes (CYP450) [28]. Doan *et al.* [29] determined that 80% of the hospitalized elderly population with polypharmacy have a potential CYP450-mediated DDI, with the prevalence of potential DDIs increasing with the number of prescriptions.

Collectively, the higher prevalence of multimorbidity and polypharmacy, combined with age-related alterations in pharmacokinetics and pharmacodynamics, makes the older population more susceptible to ADEs [30]. Polypharmacy is also associated with anxiety or excitability, sleep difficulties, discomfort, weakness, confusion, tremors, hallucinations and feeling dizzy, demonstrating a negative impact on quality of life among elderly population [31]. Besides patient’s safety, inappropriate polypharmacy poses an economic burden to healthcare system [32], due to hospital admissions or nonadherence to medications [14]. A Japanese cohort study highlighted that polypharmaceutical patients on one or more PIMs have a significantly higher hospitalization risk and more outpatient visit days, resulting in a 33% increase in healthcare cost when compared with polypharmaceutical patients on no PIMs [33]. Similar results have been found in Scandinavia, where a Swedish-based register study [34] determined that individuals on multiple medications (>5) account for 78.8% of total prescription medication acquisition costs. When compared with nonpolypharmaceutical patients, polypharmaceutical patients experienced increased prices per defined daily dose, with patients on ten or more prescribed drugs paying the most per dose [34]. Another Swedish study [35] has demonstrated that the increase in polypharmacy in recent years accounts for a large part of the increase in prescription drug expenditure. By comparing the prescription drug expenditure in 2005 and 2009, Hovstadius *et al.* [35] determined that the increase in polypharmacy between 2005 and 2009 could explain the entire increase in prescription drug expenditure in Sweden for that period. Several lists and guidelines have been developed around the world in attempt to prevent inappropriate polypharmacy and proper treatment of co-morbidities [36], including the US Beers criteria [15], Norwegian General Practice criteria [37], and Screening Tool of Older Patients’ Prescriptions (STOPP) [38–40]. A modified version of the STOPP criteria is currently in use in Denmark [39,41], which has been developed by the Danish Geriatrics Society [42].

While some older studies have investigated patterns of polypharmacy in different regions in Denmark, including Funen [43] and Jutland [44], to the best of our knowledge, no recent Danish study has examined the pattern of polypharmacy and relationship between polypharmacy and DDIs, ADEs and ADRs, with a focus on nursing homes. Therefore, the aim of this study was to identify patterns and characteristics of polypharmacy in the elderly population living in nursing homes in Aalborg municipality, Northern region of Denmark, by investigating the DDIs and potential adverse effects. We proposed that our study could provide evidence for the existence of polypharmacy and potentially inappropriate polypharmacy in the elderly population above 65 years living in these nursing homes.

## Methods

### Study population

This study was conducted in 2019 based on medication lists obtained from nursing homes in Aalborg municipality, Northern Jutland, Denmark. The target population of this study was the polypharmaceutical elderly. Elderly was defined as being over 65 years of age, while polypharmacy was defined as the concurrent use of five or more prescription-based pharmaceutical dosage forms. In this definition, the time-limited medications, such as antibiotics, were not included, since the five or more medications should be taken regularly for a longer period of time. Furthermore, vitamins and food supplements that do not need a prescription (e.g., calcium, D vitamin, multivitamin) were not included in this category; hence, they were excluded from calculation of the total number of medications taken by each patient. Supplements that require a prescription, which in this study were vitamin B12 and potassium chloride, were included.

The participants in this study were recruited in the period of 7–27 November 2019, from nursing homes located in different parts of Aalborg municipality with following postal codes: 9000 Aalborg, 9200 Aalborg south west, 9210 Aalborg south east, 9220 Aalborg east, 9270 Klarup, 9280 Storvorde, 9310 Vodskov, 9382 Tylstrup and 9400 Noerresundby. The different nursing homes were identified using a publicly available list of nursing homes in Aalborg municipality [45].

Due to the nature of this study, the ethical approval from the North Denmark Region Committee on Health Research Ethics was not required. Since participants’ information was provided anonymously by nursing homes, no consent form was required.

### Study process

**Figure 1.**
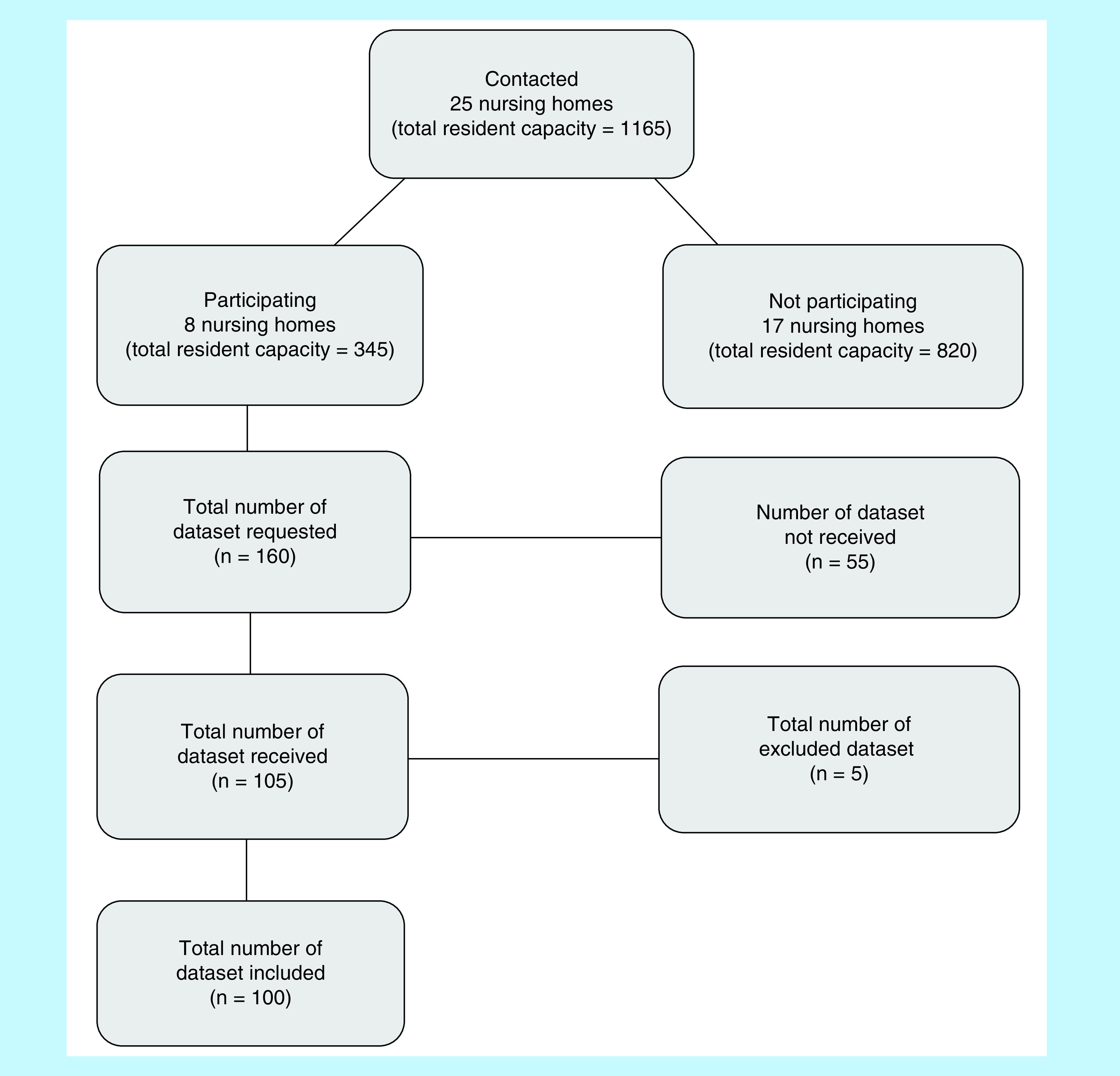
Flowchart illustrating the study steps of recruitment of the residents’ information.

In total, 25 nursing homes were identified and contacted (total resident capacity = 1165) by personal attendance in order to determine their interest in participating in this study voluntarily. Each nursing home was asked to supply the following information from 20 randomly selected residents among those aged 65 years or older, who were concurrently taking five or more medications: age, sex, co-morbidities and medication list. Each nursing staff was asked to anonymize the data for each resident before turning it over in order to comply with the general data protection regulation, which is currently in effect in the European Union. Hence, the study remained anonymous. Recruitment process is depicted in Figure 1.

When illustrating the total resident capacity, it is important to state that this number does not reflect the actual number of residents at nursing homes. The capacity number has been reported instead of the actual number of current residents, since the number of residents is continuously changing.

The nursing homes that expressed interest were given a period of 1 week to consider whether or not they wanted to participate, followed by a period of 1 week to obtain the data. Afterward, the requested information was either collected from the nursing homes in person or received by E-mail. The nursing homes that refused to participate expressed that this was due to lack of time or nursing staff, participation in other projects/studies, or other reasons.

### Data analysis

After receiving the datasets, data management was performed using Google sheet. The data were collected in three separate sheets, of which the first contained age, sex and co-morbidities; the second contained the individual medication lists; and the third contained the identified DDIs among the medications. In the first sheet, the co-morbidities were divided into following classes: ‘cardiovascular,’ ‘central nervous system,’ ‘dermatological,’ ‘endocrinological,’ ‘eyes and ears,’ ‘gastrointestinal,’ ‘musculoskeletal,’ ‘pulmonal,’ ‘psychological’ and ‘urogenital.’ The co-morbidities were indicated as numbers, which means a total number of co-morbidities was defined and recorded.

Prescription medications taken by each subject of the study were sorted in the second sheet according to their Anatomical Therapeutic Chemical (ATC) code, which has been the recommended classification system for drug utilization studies since 1981 by the WHO (Geneva, Switzerland) [46]. This categorization was performed at the second level of the ATC code, meaning that drugs were sorted according to their therapeutic subgroup (e.g., ‘A01, stomatological preparations,’ ‘A02, drugs for acid-related disorders’) [46].

The third sheet was completed utilizing two different drug interaction databases. One is run by the Danish Medication Agency [47] and the other by the US FDA [48]. Both databases were used to identify potential DDIs among the different medications. In the FDA database, the potential DDIs were divided into minor, moderate and major severities. The minor DDIs were clinically insignificant and could be used together, if a monitoring plan was made. The moderate DDIs were of modest clinical significance, these combinations should usually be avoided or only used under special circumstances. The major DDIs were highly clinically significant, whereby these combinations should be avoided [48]. In the Danish database, minor potential DDIs were those with clinical consequences known to be insignificant or nonexistent. Moderate potential DDIs were combinations of drugs that can be used therapeutically, if certain precautions are taken (e.g., dosage adjustments or changing the timing of ingestion). Major potential DDIs were combinations that are contraindicated or combinations that consisted of at least one drug with no scientifically proven effect [47]. In the database run by the FDA, Microlax (sodium lauryl sulfoacetate), Bromazepam, Flupentixol, Thiamazol, Zuclopenthixol, Moxonidin, Lercanidipine and Chlorprothixen were not available, due to the lack of approval in the USA. These drugs could not be checked for any potential interactions using the FDA database. After managing the data in Google sheets, different illustrations of data were made, including tables, graphs and pie charts.

### Statistical analysis

The following statistical tests were performed using the software IBM SPSS Statistics version 25 for Mac OS X. A statistical significance level of 0.05 and a 95% CI were used to state whether a statistical significance was present. Prior to the statistical tests, all data (age, sex, number of drugs and number of DDIs) were tested for normal distribution by using the Shapiro–Wilk test.

Three nonparametric tests were performed. A Mann–Whitney U test was utilized to compare the number of drugs and the number of DDIs among the female and male residents and the Kruskal–Wallis test was performed to compare the number of drugs and the number of DDIs among four different age groups (65–74; 75–84; 85–94; 95+). The results from both the Mann–Whitney U and the Kruskal–Wallis tests are presented as median (25–75 percentiles). A Kendall’s tau-b correlation was performed to investigate the relationship between the number of drugs and DDIs, number of drugs, age and the DDIs between the two databases.

## Results

### Demographics

One hundred (n = 100) subjects from eight different nursing homes were identified as eligible for data analysis. The mean (± standard deviation) age in this population was 82.9 ± 7.7 years and out of 100 subjects, 68% were females. This population presented on average 5.8 ± 2.2 co-morbidities, taking on average 8.5 ± 2.6 prescription-based pharmaceutical dosage forms. The demographics of these subjects are presented in Table 1.

**Table 1.** Demographic characteristics divided into four age groups. Values are expressed as number of subjects and percentages.

Characteristics	Age (years)				Total
	65–74	75–84	85–94	95+	
Population	14 (14%)	44 (44%)	36 (36%)	6 (6%)	100
Sex					
– Female	6 (8.8%)	32 (47.1%)	24 (35.3%)	6 (8.8%)	68
– Male	8 (25%)	12 (37.5%)	12 (37.5%)	0 (0%)	32
Co-morbidities					
– 0–4	4 (12.5%)	12 (37.5%)	12 (37.5%)	4 (12.5%)	32
– 5–9	10 (16.1%)	29 (46.8%)	21 (33.9%)	2 (3.2%)	62
– 10+	0 (0%)	3 (50%)	3 (50%)	0 (0%)	6
Drugs					
– 5–9	11 (16.2%)	23 (33.8%)	29 (42.6%)	5 (7.4%)	68
– 10+	3 (9.4%)	21 (65.6%)	7 (21.9%)	1 (3.1%)	32

### Co-morbidities & medications

**Figure 2.**
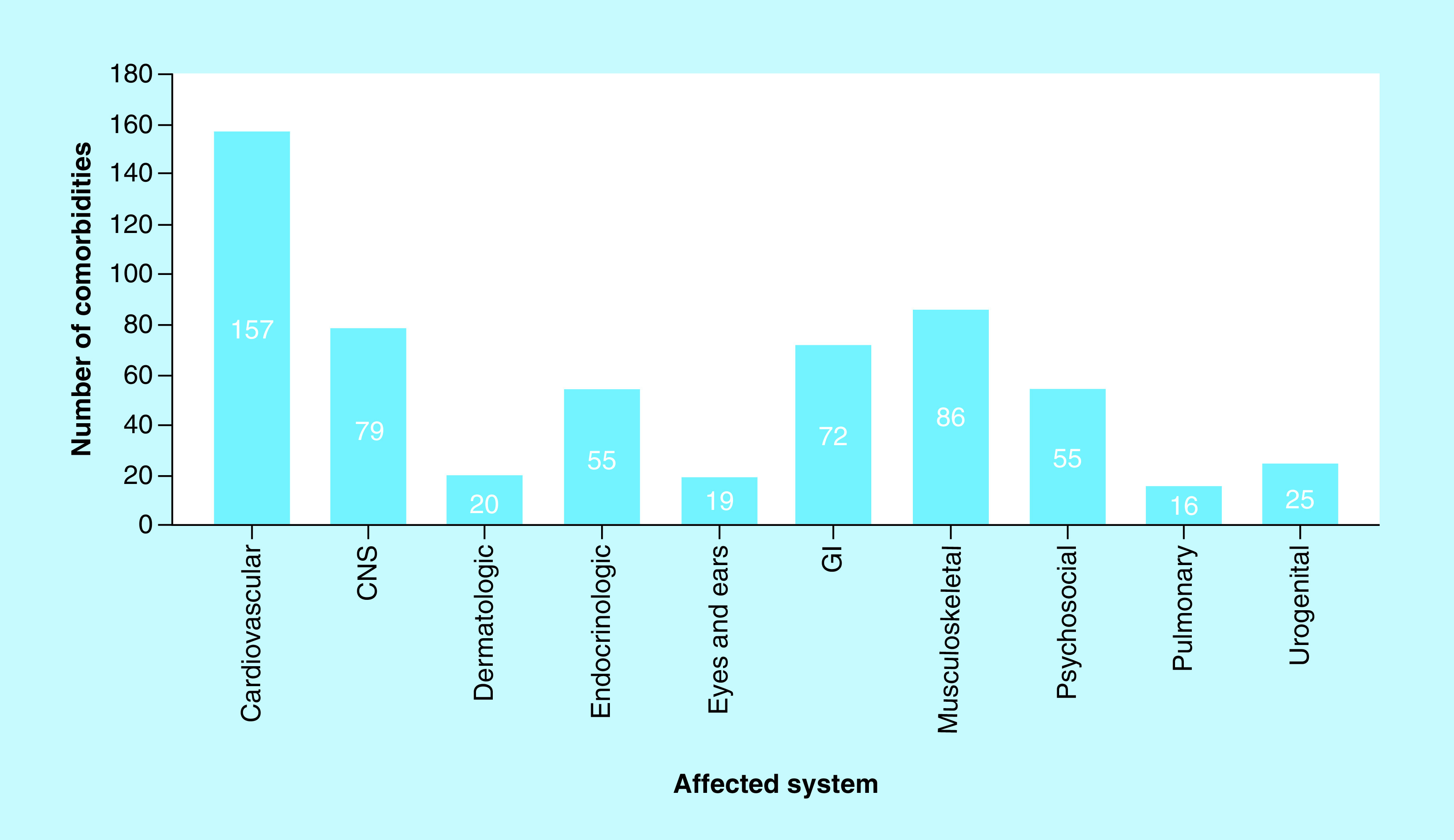
The distribution of co-morbidities by affected systems. CNS: Central nervous system; GI: Gastrointestinal.

A total of 587 co-morbidities among the 100 residents were registered. As shown in Figure 2, the cardiovascular system was the most affected system with 157 identified co-morbidities. The second most affected system was the musculoskeletal system (86 co-morbidities), followed by the CNS (79 co-morbidities).

A total of 848 prescriptions were identified and categorized according to the ATC codes. Table 2 displays the sorting of these prescription drugs into their anatomical main groups.

**Table 2.** The distribution of prescribed medications by the Anatomical Therapeutic Chemical codes.

Overall drug classes	Number of prescriptions identified	Number of patients receiving drugs	Average no. of drugs received by each subject with 1≤ active prescription
Alimentary tract and metabolism (A)	240	92	2.6
Blood and blood-forming organs (B)	64	62	1.0
Cardiovascular system (C)	191	83	2.3
Dermatologicals (D)	14	12	1.2
Genitourinary system (G)	16	14	1.1
Systemic hormonal preparations (H)	15	15	1
Anti-infectives for systemic use (J)	8	8	1
Antineoplastic and immunomodulating agents (L)	5	5	1
Muscular–skeletal system (M)	27	27	1
CNS drugs (N)	219	83	2.6
Antiparasitic products (P)	2	2	1
Respiratory system (R)	18	15	1.2
Sensory organs (S)	24	13	1.8
Other drugs^†^	5	5	1

† Other drugs include categories of drugs, which only contained one prescription. These are: digestives including enzymes (A09), antianemic preparations (B03), antibiotics and chemotherapeutics for dermatological use (D06), antivirals (J05), anti-inflammatory and antirheumatic preparations (M01).

CNS: Central nervous system.

### Drug–drug interactions

**Figure 3.**
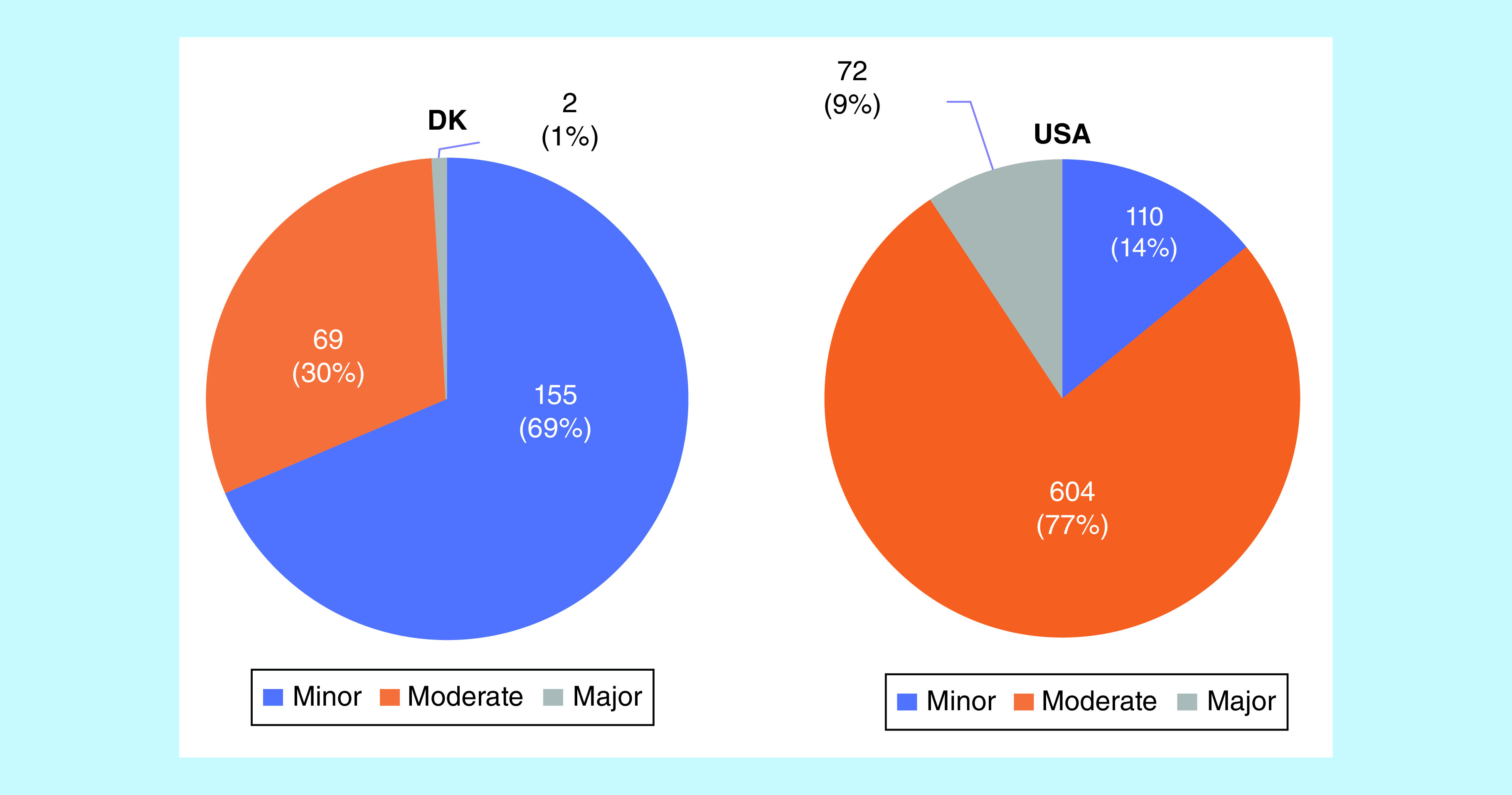
The distribution of potential drug–drug interactions found in the Danish drug interaction database (DK) and the FDA drug interaction database (USA). The DDIs were divided into three categories based on severity: minor, moderate and major. DDI: Drug–drug interaction.

Figure 3 illustrates the distribution of minor, moderate and major potential DDIs for both the FDA and Danish drug interaction databases. When inspecting the data from the Danish database (A), the minor DDIs made up the majority (68.6%) of the total number of interactions (n = 226). Comparatively, the moderate potential interactions were accountable for the majority (76.8%) of the total number of potential interactions (n = 786), when inspecting the data from the FDA database (B).

In Table 3, the ten most common DDIs were identified, of which the most common ones were Furosemide and Metoprolol (number of cases = 13). Among the ten most common DDIs, only DDIs of moderate severity were identified, which means no major DDIs were presented. Furthermore, the potential clinical effects of these DDIs are presented. The minor DDIs were not included.

**Table 3.** The ten most common moderate and major drug–drug interactions identified in the Danish and the FDA drug interaction databases. The potential clinical effects and the severity based on clinical significance are from the databases. The minor drug–drug interactions were not included.

Rank	DDI	Number of cases	Potential clinical effect	Severity
1	Furosemide + Metoprolol	13	Hyperglycemia and hypertriglyceridemia	Moderate
2	Furosemide + Pantoprazole	11	Hypomagnesemia	Moderate
3	Furosemide + Sodium picosulfate	9	Fluid and electrolyte disturbances	Moderate
4	Metoprolol + Amlodipine	9	Further reductions in heart rate, cardiac conduction and cardiac contractility	Moderate
5	Clopidogrel + Pantoprazole	8	Reduced bioactivation of clopidogrel and the therapeutic efficacy	Moderate
6	Metformin + Insulin	7	Hypoglycemia	Moderate
7	Mirtazapine + Metoprolol	7	Hypotension	Moderate
8	Furosemide + Insulin	7	Hyperglycemia, glucose intolerance, new-onset diabetes mellitus and/or exacerbation of pre-existing diabetes	Moderate
9	Furosemide + Metformin	7	Increased metformin levels potentially leading to lactic acidosis	Moderate
10	Atorvastatin + Clopidogrel	6	Reduced bioactivation of clopidogrel and its antiplatelet effect	Moderate

### Comparisons

The Shapiro–Wilk test of normality revealed no significance for co-morbidities, sex or DDIs in both the FDA and the Danish databases, whereas a significance was observed for age (p = 0.330). This means that data from sex, co-morbidities, number of drugs and DDIs did not follow a normal distribution, whereas the data from age were normally distributed.

#### Age

The results from the Kruskal–Wallis test comparing the association between number of drugs per resident and the different age groups are presented in Table 4. The results showed significant differences (p = 0.013) between the four different age groups. Furthermore, an investigation on the relation between the number of DDIs per resident and age groups was performed by using the Kruskal–Wallis test. As seen in Table 4, the highest number of DDIs per resident was observed in the age group of 75–84 years old. A statistical significance in the FDA database (p = 0.039) was revealed, however no significant difference was observed in the Danish database (p = 0.122).

**Table 4.** The median (25–75 percentiles) number of drugs and drug–drug interactions in the Danish and the FDA databases in the four different age groups.

Age (years)	Number of drugs	DDI (Danish database)	DDI (FDA database)
65–74	8 (6.5–9.5)	1 (0–4)	6 (4–9.5)
75–84	9 (8–11)	2 (1–4)	7.5 (4–13.75)
85–94	7 (6–9)	1.5 (0.25–3)	5 (2.25–7)
95+	7 (6–9)	1 (1–1)	4 (1–7)

DDI: Drug–drug interaction.

#### Sex

As seen in Table 5, there was a higher number of drugs per resident among the females compared with the males, however, this was not significant (p = 0.070). Furthermore, a Mann–Whitney U test was performed to analyze if an association exists between the number of DDIs per resident and sex, the results of which are illustrated in Table 5. No significant difference was found in the number of DDIs per resident between females and males in the FDA (p = 0.269) or the Danish database (p = 0.397).

**Table 5.** The median (25–75 percentiles) number of drugs and drug–drug interactions in the Danish and the FDA database for females and males.

Sex	Number of drugs	DDI (Danish database)	DDI (FDA database)
Female	9 (7–10)	2 (1–3)	6 (3–12)
Male	7.5 (6–9.75)	1 (0.25–3)	6 (2.25–8)

DDI: Drug–drug interaction.

### Correlations

Kendall’s tau-b performed on the number of drugs per resident and age, revealed a correlation coefficient of -0.151, which indicates a minor inverse correlation between these two variables, suggesting that as age increases the number of drugs decreases. Kendall’s tau-b was also performed on data including number of drugs and the number of DDIs per resident (both in the FDA and Danish database). The correlation coefficients were 0.400 between DDIs in the Danish database and the number of drugs, and 0.548 between DDIs in the FDA database and the number of drugs. These correlation coefficients indicate the presence of positive correlations between all variables, meaning that as one variable increases, the other also increases.

## Discussion

This preliminary study provides the first evidence of potentially inappropriate polypharmacy existing in Danish elderly resident homes, located at Aalborg municipality. Findings from this study, therefore, must be viewed as a starting point of research concerning polypharmacy in Danish elderly resident homes.

### Demographics

In the present study, 100 residents from nursing homes in Aalborg municipality were included, of which 68% were females. Females constitute 54% of the Danish elderly population [49]. The observed difference between the general elderly population and the sample population in this study could suggest that females are more susceptible to polypharmacy and thus inclusion in the study, which would be in accordance with previous findings [33,50,51]. However, one study of European nursing home residents found that 73% of a total study population of 4023 subjects were females [51]. This indicates that the high percentage of females is a reflection of the over-representation of females among nursing home residents in Europe. This might be attributed to sex-based differences in mortality, a difference that increases with age [52].

Furthermore, the mean age of the present population was 82.9 ± 7.7 years. This number is comparable to numbers found across Europe. One study determined an average age of 83.5 ± 9.4 years among nursing home residents across seven different European countries [51]. Akner’s subjects also were 85 ± 7 years of age in a nursing home in Sweden, pointing out to similar results in Scandinavia [4].

Collectively, the demographics of the subjects included in this study seem representative of Danish nursing home residents in general. This is supported by a national study conducted by the Danish Ministry of Health (Sundheds-og Ældreministeriet) [53]. They demonstrated that on average, 68% of nursing home residents in Denmark were females. While a mean age for current residents of nursing homes was not reported, they did find that the average age for new residents in nursing homes was 83.7 years of age, with residents on average living in nursing homes for 2 years and 8 months, pointing out to a true mean slightly higher than 83.7 years.

### Co-morbidities & medications

Among the residents, an average of 5.8 co-morbidities was identified by asking nursing home staff to provide information on co-morbidities present in any given subject, which they may have done in a variety of ways. This number is in contrast to the findings by Akner [4], who found an average of 16.8 ± 5.3 chronic health problems among 70 residents from a Swedish nursing home. While this difference could be due to a true difference in frequency of multimorbidity between nursing homes, it most likely reflects methodological differences. Akner [4] combined medical history, clinical examinations and patient history to assemble a more complete impression of the health status of the participants, leading to a higher sensitivity in detecting chronic diseases.

The present study identified that the most commonly affected system was the cardiovascular system, followed by the musculoskeletal system and the CNS. This is not in accordance with previous findings. Akner [4] identified that the most common co-morbidities were of neuropsychiatric, cardiovascular and gastrointestinal nature. These differences might be due to an over-representation of neuropsychiatric disorders, which were present in all 70 residents in his study [4].

The number of prescribed medications per resident in this study was 8.5 ± 2.6, of which the three most commonly prescribed medications targeted the alimentary tract and metabolism, CNS and cardiovascular system. This is more than the 6.6 ± 3.2 prescribed continuously taken medications that Akner determined among the 70 residents in a Swedish nursing home [4]. This finding most likely stems from the inclusion criteria of more than five drugs in the present study skewing the average number of medications. However, it has been demonstrated that this cutoff of more than five drugs has predictable clinical discriminatory properties [10].

Another study [6] demonstrated that the three most commonly prescribed medications were psycholeptics, cardiac medications and diuretics, which are related to the systems identified as the most common affected systems in the present study. The classification of drugs according to the ATC codes conducted by Straand *et al.* [6] confirms this by identifying the CNS as the most commonly medicated system, followed by the cardiovascular system.

An inverse correlation between age and number of drugs was detected. This finding is counterintuitive, as it is well known in the literature that advanced age and polypharmacy are associated [9,27]. However, when dividing the population into four subgroups, the comparison of the of number of drugs demonstrated a statistically significant difference. The highest number of drugs per resident, based on medians, was among 75–84 year olds, while the lowest number of drugs per resident was seen among the elderly above the age of 85 years. These findings are in line with the results from the SHELTER study [51], which demonstrated fewer cases of excessive polypharmacy (>9 drugs) in the oldest nursing home residents. It has been proposed that this inverse correlation is caused by the nature of end-of-life care. When starting a new therapeutic regimen, remaining life expectancy is weighed against time required for therapeutic effect. The risk of ADRs is also higher in this patient group. These factors in combination can lead to fewer cases of excessive polypharmacy in the oldest population [54]. There is a known association between multimorbidity and polypharmacy [3], as such it can be expected that patients living with polypharmacy have a reduced life span. This number could also reflect that patients on fewer drugs have fewer co-morbidities and consequently may live longer.

There was an obvious variation in the number of subjects and the distribution of females and males within the different age groups. The results may have been influenced by these differences between the groups. This makes it difficult to assign the observed association entirely on each age group.

Additionally, a higher number of coprescribed drugs was observed among females when compared with males. While this result was not statistically significant, the tendency is in accordance with other studies. Among others, one study by Venturini *et al.* [55] highlighted that the number of drugs was higher among females, compared with males. It has been found that among Finnish and Norwegian elderly, women are slightly more likely to seek primary healthcare, which might partly explain the difference between the sexes [56]. Females also tend to report significantly more morbidity than their male counterparts, a difference that may, in part, be accounted for by gynecological and obstetrical diagnoses [57]. Accordingly, part of the difference between men and women can be attributed to the use of contraceptive pills and hormones for menopause [58]. While the sample included in this study was above 65 years, this use of contraceptives might mean that women are more accustomed to the use of drugs throughout their life and into old age [55].

### Drug–drug interactions

In the present study, when the Danish interaction database was used, 226 potential DDIs were identified, of which most were with the nature of minor severity. In contrast, when the FDA database was used, 786 potential DDIs were identified, of which most were with the nature of moderate severity. This indicates that the FDA database had a higher sensitivity for detecting DDIs when compared with the Danish database. One explanation for the difference between number and severity of potential DDIs reported by the different DDI databases may be that these databases have different physiological foci. Interaktionsdatabasen.dk is based mainly on pharmacokinetic studies, while the interaction checker on Drugs.com is based on both pharmacodynamic and pharmacokinetic studies. For this reason, there might be some pharmacodynamic DDIs that are not included by the Interaktionsdatabasen.dk. Furthermore, Interaktionsdatabasen.dk is based on data from PubMed and Embase, while Drugs.com is based mainly on data from the American medical information suppliers. Therefore, while the sensitivity was higher when using Drugs.com, the fact that the data are based on extraction from a more heterogeneous population, raises questions about the specificity of the results when applied to the very homogenous Danish population in this study. In fact, it is a known problem that different DDI screening tools offer different sensitivity and specificity. Furthermore, it has also been reported that DDI databases under-report clinically relevant DDIs and in some cases, over report insignificant drug interactions [59,60].

Combining data from both databases, the most commonly identified DDIs were Furosemide and Metoprolol, followed by Furosemide and Pantoprazole. To the best of our knowledge, no comparable, recent studies have investigated patterns of DDIs in polypharmaceutical nursing home residents. This means that when comparing the results of the present study, it is important to note that other studies may have dissimilar study populations. The identified DDIs were not in accordance with the findings from the study by Sönnerstam *et al.* [61], who found the most common clinically relevant DDI to be Furosemide and Citalopram, followed by Acetylsalicylic acid and Citalopram.

The highest number of DDIs per resident was observed in the age group 75–84 years. No significant relation was found between age groups and DDIs in the Danish interaction database, whereas a significant difference in DDIs was determined between age groups in the FDA database. This could be due to the majority of the patients in this age group (75–84 years) taking a higher number of medications, which can increase the risk of DDIs. Goldberg *et al.* [62] highlighted that for patients taking two medications, the risk of DDIs was 13%, compared with 38% for patients taking five medications and 82% for patients taking seven medications or more.

Additionally, the correlation coefficient comparing the number DDIs per resident in the Danish database and the number of drugs, indicated a positive correlation (0.400), whereas the correlation coefficient from the comparison of the number of DDIs per resident in the FDA and the number of drugs indicated a stronger positive correlation (0.548). These correlations are in accordance with the results from the aforementioned study, which demonstrated an elevated risk of DDIs when increasing the number of drugs [62]. These findings could be further explained by the fact that the amount of potential pairwise interactions increases exponentially with each coprescribed drug [63].

No significant difference was observed in the total number of DDIs among males and females in the FDA or the Danish database. These findings are supported by the medians of DDIs in both males and females, which are nearly identical. When considering the literature, this finding is unexpected. A study by Venturini *et al.* [55] reported that females are more likely to take a higher number of medications than men, putting them at higher risk for potential DDIs. A summary of our findings compared with other studies is presented in Table 6.

**Table 6.** Summary of the present findings in comparison with previous studies in the literature.

Our findings	Others findings
68% of elderly at nursing homes with polypharmacy are females	58% of the population above 65 years living at nursing homes in Denmark are females [49]
Mean (±SD) age = 82.9 ± 7.7 years	In Europe [51], the mean age is 83.5 ± 9.4 years In Sweden [4], the mean age is 85 ± 7 years New resident average age in Denmark [64]: 83.7 years
5.8 co-morbidities in average	Akner [4] found 16.8 ± 5.3 in average
8.5 ± 2.6 number of medications in average	Akner [4] found 6.6 ± 3.2 in average

SD: Standard deviation.

### Clinical consequences of DDIs

Information regarding documented clinical consequences for the residents included in this study was not available. Therefore, we reviewed theoretical and clinically observed adverse effects for the ten most commonly identified potential DDIs in the present study. Table 7 summarizes the findings.

**Table 7.** Summary of drug–drug interaction and clinical consequence.

Drug–drug interaction	Clinical consequence
Furosemide + Metoprolol	Hyperglycemia/hypertriglyceridemia
Furosemide + Pantoprazole	Hypomagnesemia
Furosemide + Sodium picosulfate	Fluid and electrolyte disturbances
Metoprolol + Amlodipine	Reduction in HR, cardiac conduction and contraction
Clopidogrel + Pantoprazole	↓ Bioactivation of clopidogrel → No antiplatelet eff.

HR: Heart rate.

The mechanism behind DDIs between Furosemide and Metoprolol is not well known; however, studies suggest that both β-blockers and loop diuretics can alter the lipid profile by elevating levels of triglycerides [65,66]. β-Blockers may also contribute to the development of hyperglycemia by diminishing the release of insulin from pancreatic β-cells [65,67]. Based on the findings of this study, interaction between these two was on top of the list of the identified DDIs; therefore, caution should be taken when prescribing these two drugs. Further studies are required to elucidate the potential clinical consequences of using Furosemide and Metoprolol.

The second most common DDI identified in this study was between Furosemide and Pantoprazole. Pantoprazole and other proton-pump inhibitors have shown to cause hypomagnesemia [67]. Urgent clinical consequences of hypomagnesemia include tremors, convulsions, delirium and even coma [68]. Since hypomagnesemia is also a side effect of Furosemide [69], the coadministration of these two drugs without taking the necessary precautions to maintain a normal serum magnesium concentration could be problematic.

Furthermore, a DDI between Furosemide and Sodium picosulfate was identified. Sodium picosulfate should be administered with caution when combined with medications that can affect renal function (e.g., Furosemide), since it may result in an increased risk of fluid and electrolyte disturbances, which may lead to seizures, arrhythmias and prolonged QT interval [70]. Another identified potential DDI was Metoprolol and Amlodipine. The combined treatment with β-blockers and calcium channel blockers has additive effects on heart rate, cardiac conduction and cardiac contractility. Cases have been documented on concurrent treatment with these two classes of drugs leading to congestive heart failure, hypotension, bradycardia or even cardiogenic shock [71,72]. We identified that potential DDIs were common when Clopidogrel was used with Pantoprazole, and when Clopidogrel was used with Atorvastatin. The conversion of Clopidogrel into its active metabolite by CYP450 isoenzymes in the liver is necessary for Clopidogrel to exert its inhibitory effect on platelet aggregation [73]. Pantoprazole, and other proton-pump inhibitors have been demonstrated to inhibit CYP450 2C9 and 2C19 *in vitro* [74], while Atorvastatin inhibits CYP450 34A [73]. Since CYP450 2C19 and 3A4 play a major role in Clopidogrel bioactivation, the coadministration of these drugs can reduce the activation and subsequent antiplatelet effect of Clopidogrel [73]. One study by Juurlink *et al.* [75] has shown an increase in the risk of recurrent myocardial infarction when treating with proton-pump inhibitors and Clopidogrel, simultaneously. However, this was not the case for Pantoprazole; hence, the clinical significance is yet to be fully established. Nonetheless, Doan *et al.* [29] observed that 80% of the hospitalized elderly population with polypharmacy had a potential CYP450-mediated DDI, thus, it is essential to be mindful when prescribing multiple drugs that interact with CYP450.

In this study, three of the ten most frequent DDIs included an antidiabetic drug. These DDIs included Metformin and Insulin, Metformin and Furosemide, and Insulin and Furosemide. Both Metformin and Insulin can lead to hypoglycemia [76], which is the most common side effect of glucose-lowering drugs in general [76]. Metformin is regarded as a low-risk drug for hypoglycemia, since it exerts its effect through a glucose-dependent mechanism [76]. However, several studies have observed an evident association between the use of insulin and higher risk of hypoglycemia [76]. A hypoglycemic episode leads to an activation of the autonomic nervous system resulting in minor symptoms, from sweating and palpitations, to more severe symptoms such as cognitive dysfunction and seizures. Depending on the severity and duration of the episode, hypoglycemia can even result in coma or death [76]. Considering these possible detrimental side effects of glucose-lowering drugs, in particular insulin, it is important to cautiously manage the treatment of a patient whose conditions require both types of drugs.

When using Furosemide in combination with Metformin, Furosemide increases the plasma concentration level of Metformin by 22% [77]. The clinical implications of this increase are uncertain [77], but it may be possible that it can result in lactic acidosis, which is a known reaction to Metformin [78]. When the plasma concentration of Metformin increases, the risk of this reaction could also increase.

The combination of Furosemide and Insulin can exert an inhibitory effect on the secretion of Insulin from the β-cells of the pancreas, which can generate a problem for patients receiving Insulin as medication as the patient’s dose takes endogenous insulin production into account. This can result in hyperglycemia, due to the overall lower amount of insulin circulating in the blood. To account for this interaction, a dose adjustment should be considered [79].

Since several of the most common DDIs included antidiabetic drugs, it should also be noted that people with Type 2 diabetes mellitus are often of advanced age [80] and at a higher risk of polypharmacy, which makes them more exposed to potential DDIs and side effects [81].

A potential DDI between Metoprolol and Mirtazapine was identified. Newer antidepressants are known to have cardiovascular side effects [82]. As such, coadministration of Mirtazapine with the β-blocker Metoprolol would presumably lead to additive hypotensive effects, as well as changes in pulse and/or heart rate. However, it has been observed that coadministration of Metoprolol and Mirtazapine is safe with no clinically significant DDIs [83]. In fact, this lack of clinical adverse effects has been documented to such a degree that Mirtazapine has been used as the negative control in a case–control study of interactions between Metoprolol and selective serotonin reuptake inhibitors [84].

The most common, potentially clinically relevant DDIs identified in this study were of moderate severity; however, major potential DDIs also occurred. Three of them consisted of an interaction between opioids and benzodiazepines (Codeine and Oxazepam, Codeine and Zolpidem, and Morphine and Oxazepam). The coadministration of opioids and benzodiazepines is associated with an increased risk of overdose deaths and emergency department visits [85]. Benzodiazepines have also been demonstrated to exert an exacerbating effect on opioid-mediated respiratory depression [85]. Additionally, a study has noted an elevated fracture risk among older men who are treated with a combination of opioids and benzodiazepines [86]. The FDA recommends that healthcare professionals minimize the prescription of opioid medications with benzodiazepines and other CNS depressants, when possible.

### Strengths & limitations of the present study

#### Study population

This is a preliminary study with a relatively small-sample size (n = 100). Nevertheless, certain measures were taken to assure that the study population was as representative of the background population as possible. For instance, 5–20 randomized residents were identified by nurses from each of the eight nursing homes, instead of including a higher number of residents from only a few nursing homes. Furthermore, the participating nursing homes were of various geographical locations within Aalborg municipality. These approaches provided an advantage in terms of minimizing possible selection biases and confounders and enhance generalizability. However, in future studies with larger cohorts, considering both internal and external sources of bias must be taken into consideration. According to a census conducted by Aalborg municipality in 2019, 1860 nursing home residencies are currently available in Aalborg municipality. The prevalence of polypharmacy in the elderly has been estimated, by Scandinavian studies, range from as low as 16.8–30% within the range of 65–74 years to 33–65% at 95+ years [9,27]. Similar results have been highlighted by systematic review on the prevalence of polypharmacy by the WHO, where the estimated prevalence was 38.1–91.2% among nursing homes or long-term care facility residents. Our study did not aim at finding the prevalence but was a purely descriptive study. In future studies, sample size might be estimated if prevalence is the purpose of the study.

#### Lack of comparison

This study did not include a comparison to the rest of the nursing home residents, who received less than five medications. As far as the aim of this study was to elucidate the patterns and characteristics of polypharmacy, primarily related to DDIs, the inclusion of these residents with no polypharmacy did not seem relevant. This is because the amount of potential pairwise interactions increases exponentially with each coprescribed drug [63], making comparisons irrelevant. However, it is known that polypharmacy is associated with increased age, multimorbidity and female sex [3,27,50]. The lack of this comparison in the present study makes it impossible to determine whether the demographics of the study population differ significantly when compared with a nonpharmaceutical background population. For example, the female population of this study was 68%. It is difficult to conclude what this number reflects without a comparable group from the same population (elderly living without polypharmacy in nursing homes in northern Jutland). While 68% of the population living in nursing homes in Denmark are females, it cannot be ruled out that a lower proportion of nursing home residents are females in Aalborg, with this study including these females to a higher degree.

#### Classification of drugs

By using the ATC codes, promoted by the European Medicines Agency to categorize medications, this study has tried to utilize a reliable system of drug classification in order to improve reproducibility and transparency. However, due to the lack of access to information regarding the general health status of the subjects, the categorization of drugs into therapeutic subgroups was complicated. Because a single drug can have different ATC codes depending on the therapeutic purpose [46] and lack of knowledge surrounding current health problems might have led to the condition that some drugs may have been categorized with a bias.

#### Definition of polypharmacy

Only prescription medications were included in this study, even though herbal and dietary supplements (HDS) can interact with prescription-based drugs [87,88]. Food supplements are defined as ‘foodstuffs the purpose of which is to supplement the normal diet and which are concentrated sources of nutrients or other substances with a nutritional or physiological effect, alone or in combination, marketed in dose form’ (The European Commission [89]). Because of this broad definition of food supplements alone, and the subsequent difficulty of defining and including all potentially relevant HDS, it was decided that HDS would be kept out of the scope of this study. However, due to the importance and practical benefits of such studies, it is recommended for a future investigation. This is mainly because the use of HDS is on the rise [90]. Cases have even been reported of so-called ‘polyherbacy,’ implying similarity to those associated with polypharmacy. It has been demonstrated that HDS–drug interactions can lead to adverse clinical outcomes such as hospitalizations [91]. Since an increase in the number of prescription drugs leads to an increased risk of DDIs [17], it would be rational to consider that polypharmaceutical patients are also at a higher risk of HDS–drug interactions. This suggests that there are possibly some potentially clinically relevant HDS–drug interactions that were not detected in this study. Considering the possible clinical significance of these interactions, it is relevant to include HDS in future studies to acquire a thorough understanding of potential problems related to polypharmacy in elderly.

## Conclusion

The findings from this study suggest that potentially inappropriate polypharmacy exists among elderly nursing home residents in Aalborg municipality. A direct correlation was determined between the number of drugs and number of DDIs. A complex relationship between age and number of drugs became evident, where age was inversely correlated with number of drugs. A significant difference in number of drugs was observed between the different age groups, where the highest median number of drugs was determined in the age group of 75–84 years, whereas the lowest medians of concomitant drugs were seen in the oldest age groups. In line with this finding, significant differences in the number of potential DDIs were observed in the FDA database between the different age groups. Tendencies were observed with higher number of drugs and more potential DDIs in females compared with males. The clinical consequences of the ten most commonly observed potential DDIs in this study consisted of hyperglycemia, hypotriglyceridemia, hypomagnesemia, fluid and electrolyte disturbances.

## Future perspective

Polypharmacy in the elderly is still under recognized. There is a need for awareness and identification of cases with inappropriate polypharmacy among elderly. Identification of elderly residents in nursing homes with this condition can help determine better prevention strategies to enhance the quality of life of these individuals. Our findings call for further investigation and development of guideline strategies. Establishing a detailed understanding of the patterns and characteristics of potential inappropriate polypharmacy in the elderly could provide a foundation for minimizing health and economic consequences caused by inappropriate polypharmacy. To achieve this, a larger scale study is proposed – collecting sample data from different regions in Denmark and including a control group, the inclusion of factors such as diet, dietary supplements and consumption of alcohol and tobacco would also yield further information. Inclusion of medical records would make it possible to determine the incidence rate of the potential DDIs leading to actual clinical consequences. As it has also been highlighted by multiple Danish studies on polypharmacy, the current state of polypharmacy in Denmark calls for more thorough medication reviews in the elderly [92,93], for example, through introduction of the STOPP criteria [38–40] on a broader scale.

**Summary points**

- Polypharmacy is the concurrent use of more than one drug. Multimorbidity is common among the elderly and a leading factor of polypharmacy and its risks. Since the elderly population has grown tremendously, the frequency of polypharmacy can also be expected to increase. Thus, it is important to investigate this phenomenon to prevent inappropriate polypharmacy.
- Based on the current literature, it is difficult to assess how widespread inappropriate polypharmacy is in Denmark.

**Aim**

- To identify patterns and characteristics of polypharmacy in the elderly living in nursing homes in Aalborg municipality, Denmark, by investigating the drug–drug interactions (DDIs) and potential adverse effects.

**Methods**

- Nursing homes in Aalborg municipality (n = 25) were contacted in November 2019 and asked to supply information from 20 randomly selected anonymized residents, among those aged 65 years or older, who were concurrently taking five or more medications. The collected information consisted of age, gender, co-morbidities and medication list.
- Two different drug interaction databases (one run by the Danish Medication Agency and the other by the US FDA) were utilized to identify potential DDIs among the medications taken by the residents and to divide DDIs into three levels of severity based on clinical significance.
- Descriptive statistics were applied.

**Results**

- The total number of nursing home residents included in this study was 100 (68% females; 32% males; mean age ± standard deviation [SD]: 82.9 ± 7.7 years).
- Five hundred and eighty-seven (mean ± SD: 5.8 ± 2.2) co-morbidities were registered with the cardiovascular system as the most commonly affected system. The total number of medications was 848 (mean ± SD: 8.5 ± 2.6) with the most common being medications related to the gastrointestinal system and metabolism.
- Based on the FDA database, the majority of observed potential interactions were of moderate severity (n = 786, 76.8%), whereas the Danish database identified the majority as being of mild severity (n = 226, 68.6%).
- Statistically significant differences in the number of drugs (p = 0.013) and number of DDIs per resident in the FDA database (p = 0.039) were observed among the four age groups (65–74, 75–84, 85–94, 95+). No sex-related difference was found.
- The number of drugs and DDIs were positively correlated.

**Discussion & conclusion**

- The findings suggest that potentially inappropriate polypharmacy exists among the studied elderly residents.
- Further research is needed to find if this small scope study could similarly reflect characteristics of polypharmacy in Denmark.
- Strategies and guidelines to prevent inappropriate polypharmacy in nursing homes are warranted.
